# Two-layer quality control protects Arabidopsis from transcriptional errors under heat stress

**DOI:** 10.1093/plcell/koaf264

**Published:** 2025-11-03

**Authors:** Yu-Hung Hung

**Affiliations:** Assistant Features Editor, The Plant Cell, American Society of Plant Biologists; Spearhead Bio, St. Louis, MO 63132, USA

As the world warms up due to climate change, record-shattering heatwaves have scorched many parts of the world, causing the death of thousands of people (Witze 2022). Extreme heat events also pose a threat to plant viability and agriculture. Plants must engage robust resilience mechanisms, including maintaining transcription fidelity, as faulty transcripts can lead to detrimental protein production.

In a recent study, Henrik Mihály Szaker and colleagues (Szaker et al. 2025) investigated the interplay between 2 distinct quality control mechanisms—the nuclear transcription factor TFIIS (RNA polymerase II associated elongation cofactor) and the cytoplasmic Nonsense-mediated mRNA decay (NMD) pathway—revealing that their cooperative action safeguards the transcriptome and is critical for developmental regulation and heat stress adaptation in Arabidopsis.

Using circle-sequencing (CirSeq), a reliable method for transcriptional error estimation, Szaker et al. analyzed the error landscape of RNA Polymerase II (RNAPII)-dependent transcripts in Arabidopsis. They found that transcriptional fidelity is compromised under high temperature conditions, in which the frequency of nucleotide misincorporations and insertions is significantly elevated (Szaker et al. 2025).

Szaker et al. established that TFIIS, which accelerates the excision of misincorporated nucleotides, functions as a crucial fidelity factor for transcription. In plants lacking TFIIS (*tfIIs-1* mutant), the total error rate increased 2.5-fold under normal conditions and was further elevated by 5.1-fold during heat stress compared with wild-type controls. TFIIS primarily prevents substitution errors. Furthermore, TFIIS proved essential for survival when transcription was destabilized by nucleotide imbalance stress, such as treatment with mycophenolic acid, which depletes GTP and causes misincorporations.

Despite TFIIS-mediated proofreading, some faulty transcripts are still exported to the cytoplasm, where the NMD pathway acts as a secondary surveillance system. NMD recognizes and degrades aberrant mRNAs containing premature termination codons (PTCs) (Kurosaki et al. 2019; Luha et al. 2024). Transcription errors, specifically 1 to 2 nucleotide indels (insertions or deletions), frequently cause frameshifts that generate these NMD-eliciting PTCs. Szaker et al. demonstrated that NMD specifically eliminates transcripts carrying frameshift-causing indels within the coding sequence but does not significantly alter the accumulation of substitution error–containing mRNAs under heat stress (Szaker et al. 2025).

The interdependence of these quality control systems was demonstrated through genetic analysis (Szaker et al. 2025). While single NMD mutants (*upf1-5* and *upf3-1*) show minor to strong developmental alterations, their combination with *tfIIs-1* results in severely intensified phenotypes, including stunted growth and failure to flower (in *tfIIs;upf3* mutants). Critically, the double mutants displayed synergistic and significantly enhanced sensitivity to heat stress compared with single mutants, confirming that NMD is required for heat stress tolerance. This synergy arises because TFIIS is also required for proper alternative splicing (AS), and its absence generates a large number of AS isoforms. A large portion of these TFIIS-dependent AS products bear PTCs, often due to significant lengthening of the 3'UTR or intron retention events, making them prime targets for NMD. When a plant loses both TFIIS and NMD, these aberrant transcripts accumulate, leading to enhanced proteotoxicity and severe heat sensitivity (Szaker et al. 2025).

Szaker et al. concluded that TFIIS and NMD act cooperatively: TFIIS provides the dominant nuclear quality control layer, ensuring high fidelity during RNAPII transcription and proper splicing; NMD provides the cytoplasmic backup control layer, clearing residual harmful transcripts generated by errors or deficient splicing. This 2-layer mechanism ensures transcriptome quality, which is fundamental for proper development and adaptation to high temperatures (Fig.).

**Figure.**
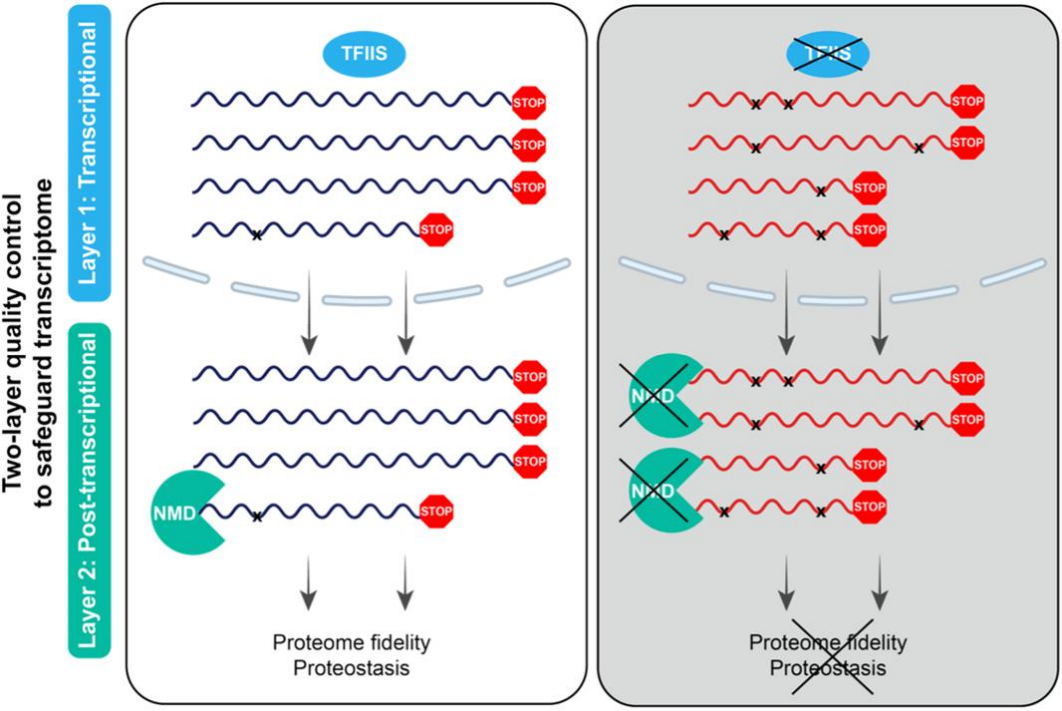
TFIIS and NMD pathways cooperate to safeguard the transcriptome under heat stress. TFIIS provides nuclear fidelity and proper splicing, primarily during RNAPII transcription, while NMD provides post-transcriptional cytoplasmic cleanup of faulty transcripts, crucial for proteostasis and heat stress survival. Modified from Szaker et al. (2025), Figure 8D.

## Recent related articles in *The Plant Cell*

- (Cai et al. 2024) identified ECT8 as an m^6^A reader protein. ECT8 acts as an abiotic stress sensor, facilitating mRNA decay to maintain transcriptome homeostasis under abiotic stress.
- (Jo et al. 2025) demonstrated that Arabidopsis PP2A B′η dephosphorylates key spliceosome components to regulate alternative splicing of heat stress–responsive genes, thereby promoting thermotolerance during seed germination.

## Data Availability

No new data were generated or analysed in support of this research.

## Dive Curated Terms

The following phenotypic, genotypic, and functional terms are of significance to the work described in this paper:

TFIIS Gramene: AT2G38560
TFIIS Araport: AT2G38560

ECT8 Gramene: AT1G79270
ECT8 Araport: AT1G79270

GTP CHEBI: CHEBI:15996
